# Supplementary material for: Molecular sociology of virus-induced cellular condensates supporting reovirus assembly and replication
Source: Nat Commun. 2024 Dec 6;15:10638. doi: 10.1038/s41467-024-54968-7 (PMC11621325; doi:10.1038/s41467-024-54968-7)
Supplement: Supplementary file 1 — Supplementary Information [file 41467_2024_54968_MOESM1_ESM.pdf]

# Supplementary Information

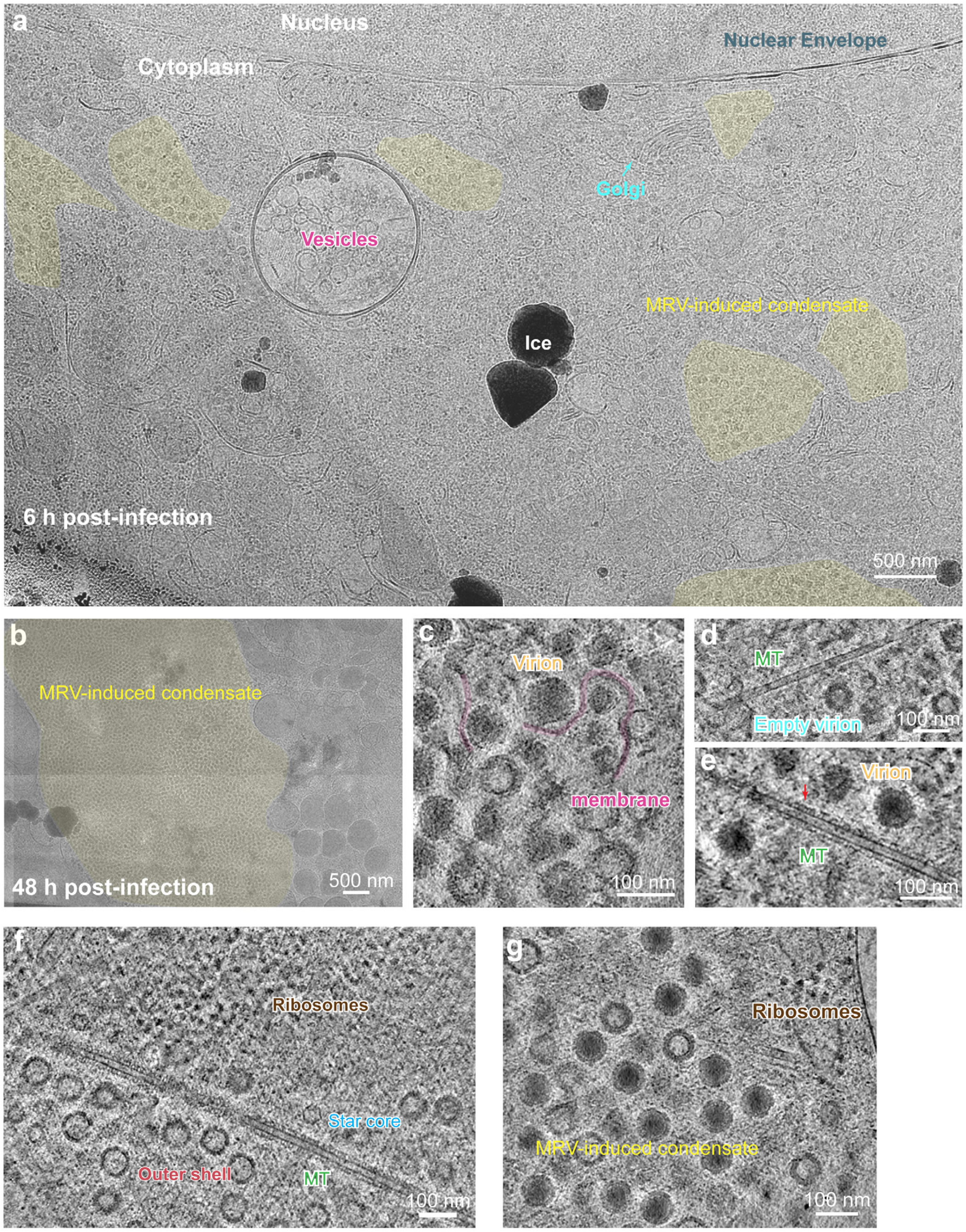


**Fig. S1: MRV-induced cellular condensates captured by cryoET. a**, Low magnification cryoEM image of the lamella from cells at 6 h post-infection. **b,** Low magnification cryoEM image of the lamella from cells at 48 h post-infection. **c-g**, Slice images from cryoET reconstructions showing different assembly intermediates and their interplay with host cell components, such as ribosomes, membranes, and microtubules (MT). The undecorated microtubule is shown in (d), and the decorated one is shown in (e). The decoration is indicated by a red arrow.

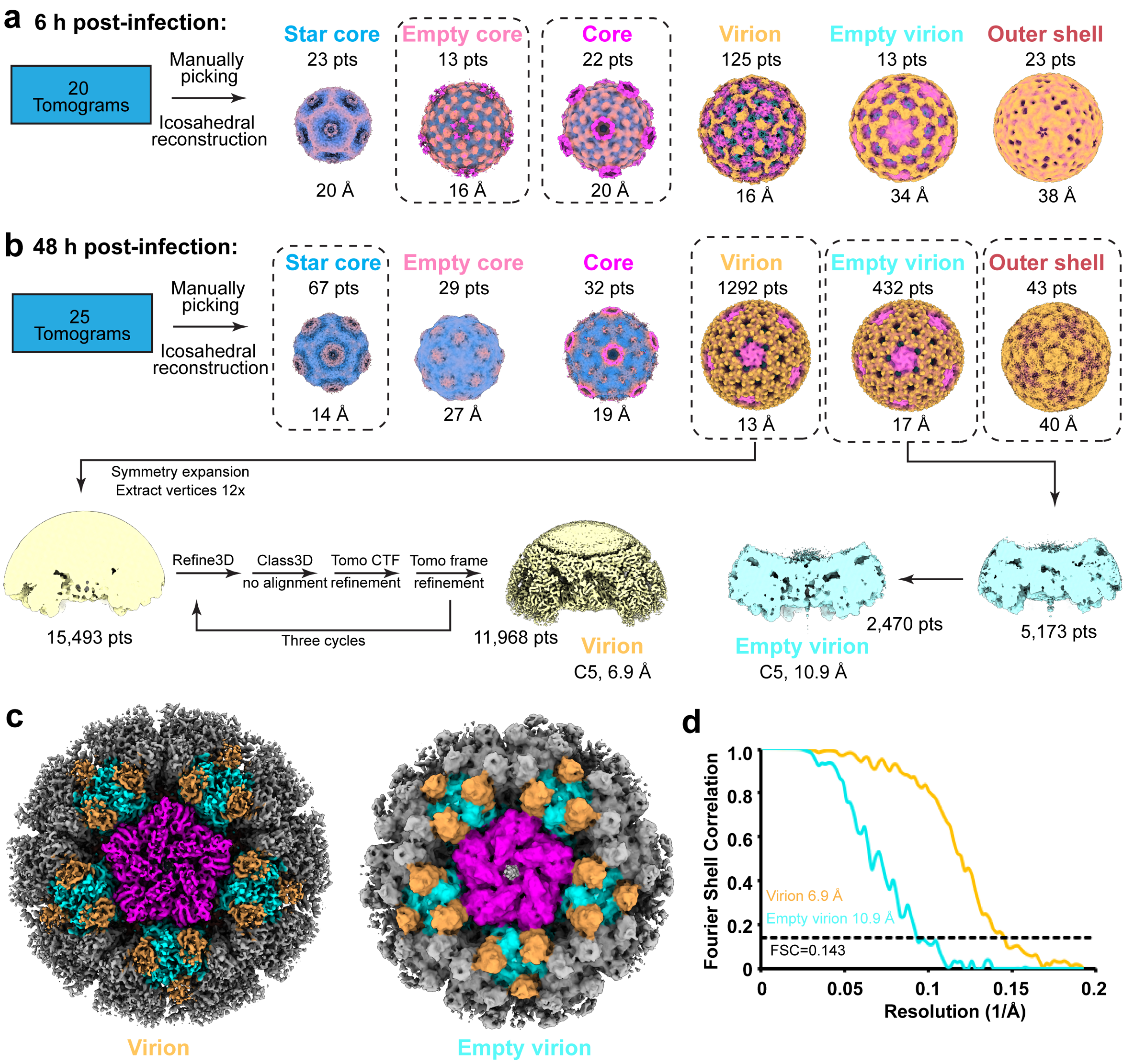


**Fig. S2: In situ cryoET Data processing.** **a**, Workflow of subtomogram averaging of cryoET data from cells at 6 h post MRV infection. Six assembly intermediates are captured. **b**, Workflow of subtomogram averaging of cryoET data from cells at 48 h post MRV infection. Six assembly intermediates are captured. Virion and empty virion were subjected to sub-particle reconstruction to improve resolution. Icosahedral reconstructions are colored by radius in a and b. **c**, Density map of virion and empty virion after sub-particle reconstruction from (b). **d**, Gold-standard Fourier shell correlation (FSC) curves for subtomogram averaging of virion and empty virion from (b).

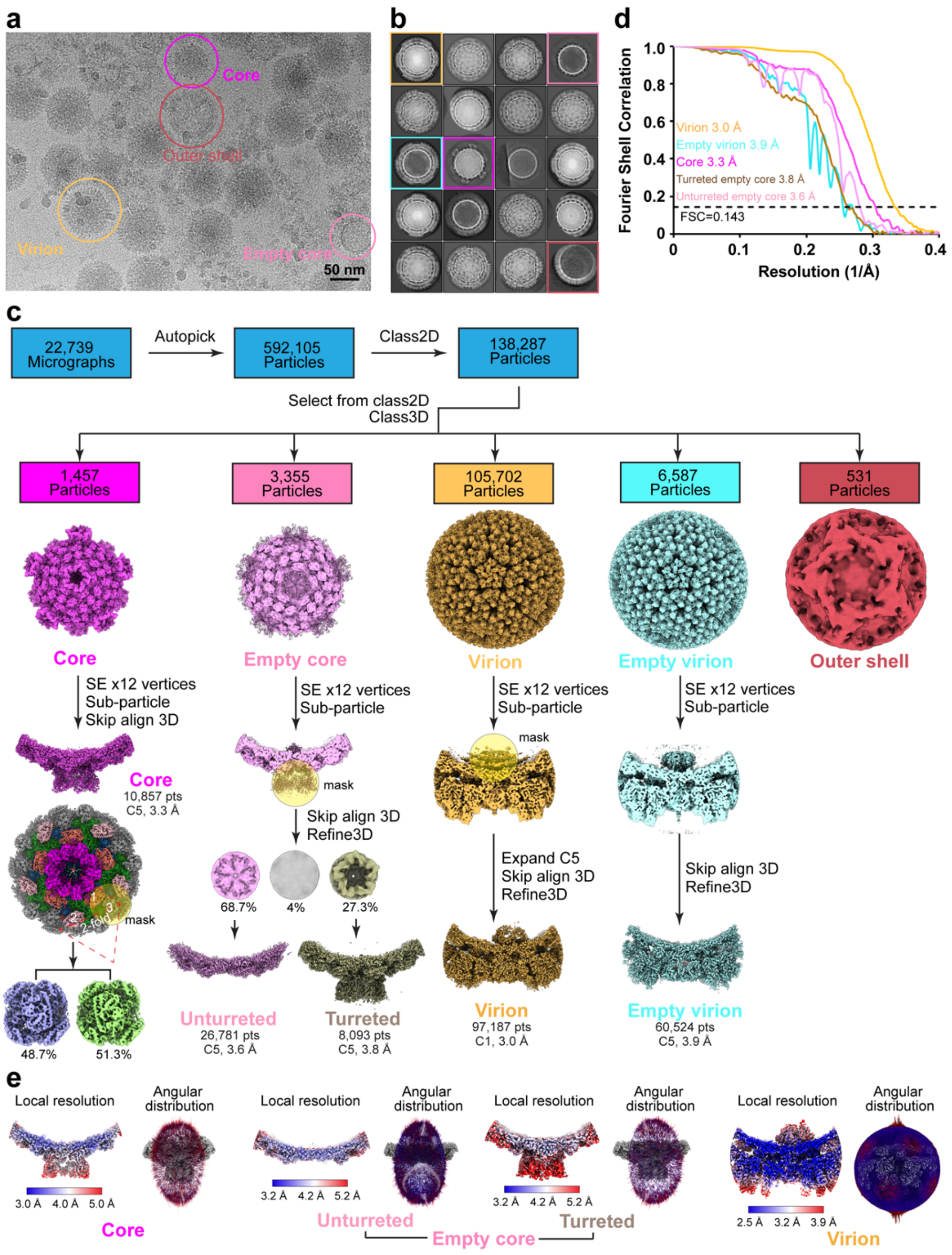


**Fig. S3: CryoEM data processing of the MRV viral isolate from host cells at 6 h post-infection. a**, Representative cryoEM image of viral isolate from host cells. Different assembly intermediates are indicated by circles and labelled. **b**, Selected 2D class averages of the cryoEM particles. Colored boxes showing different assembly intermediates. **c,** Flow chart of cryoEM data processing showing five assembly intermediates. Yellow circles on sub-particle reconstructions of core, empty core and virion show the masked area of focused classification. **d**, Gold-standard Fourier shell correlation (FSC) curves for cryoEM maps from sub-particle reconstructions. **e**, Maps colored to local resolution and representations of the angular distribution of particles used in the final reconstruction.

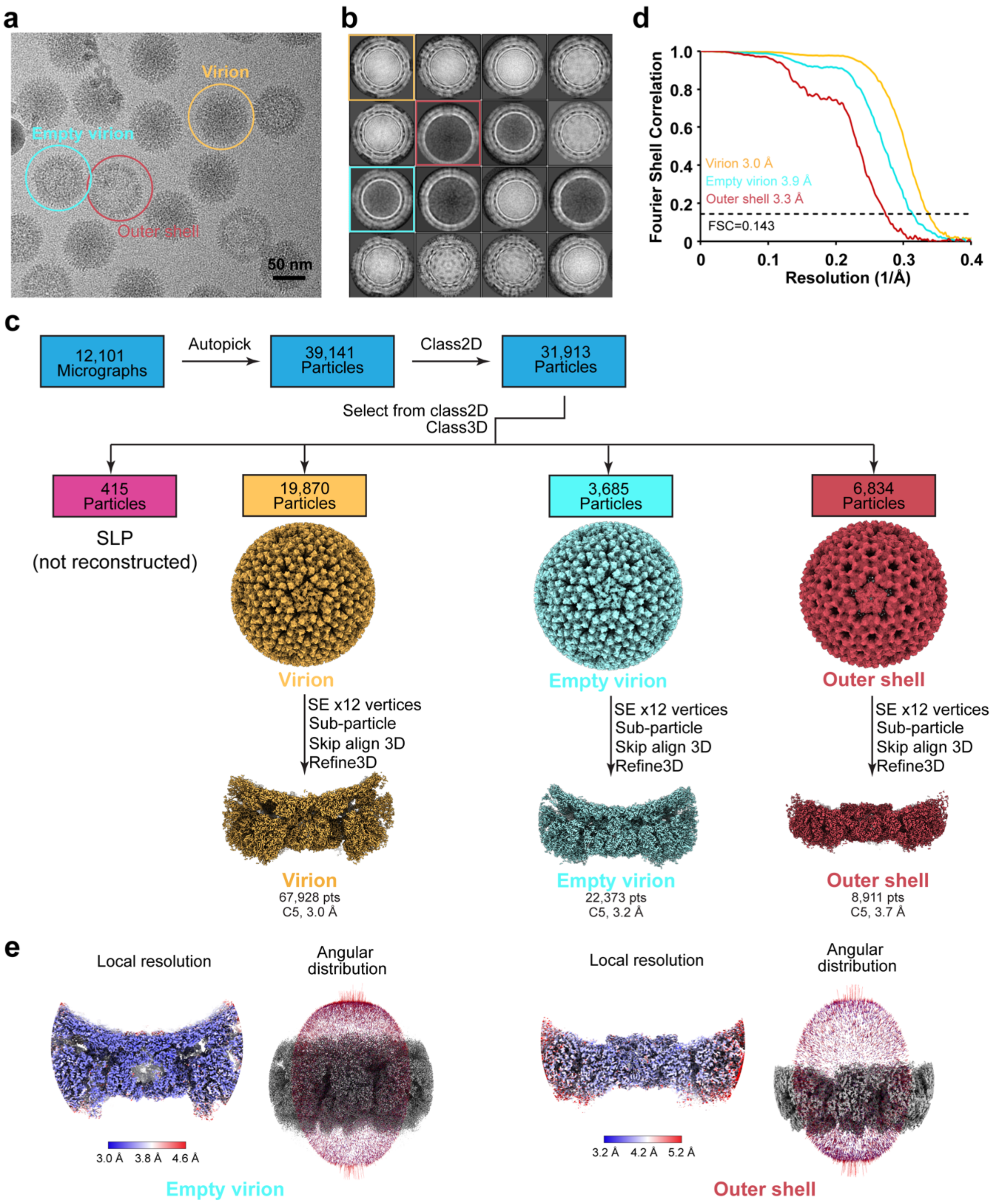


**Fig. S4: CryoEM data processing of the MRV viral isolate from host cells at 48 h post-infection. a**, Representative cryoEM image of viral isolate from host cells. **b**, Selected 2D class averages of the cryoEM particles. Colored boxes showing different assembly intermediates. **c,** Flow chart of cryoEM data processing showing three assembly intermediates. **d**, Gold-standard Fourier shell correlation (FSC) curves for cryoEM maps from sub-particle reconstructions. **e**, Maps colored to local resolution and representations of the angular distribution of particles used in the final reconstruction.

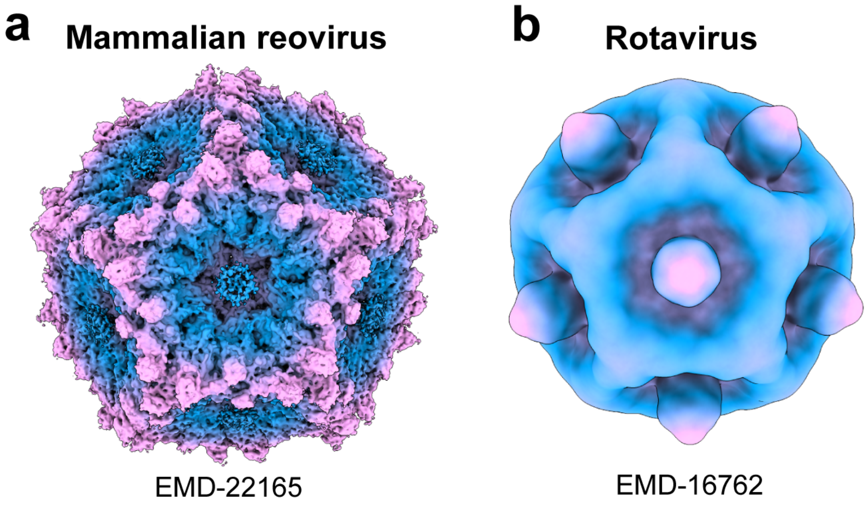


**Fig. S5: Star-shaped SLP of mammalian reovirus (MRV) and rotavirus from previous studies.** CryoEM maps are colored by radius.

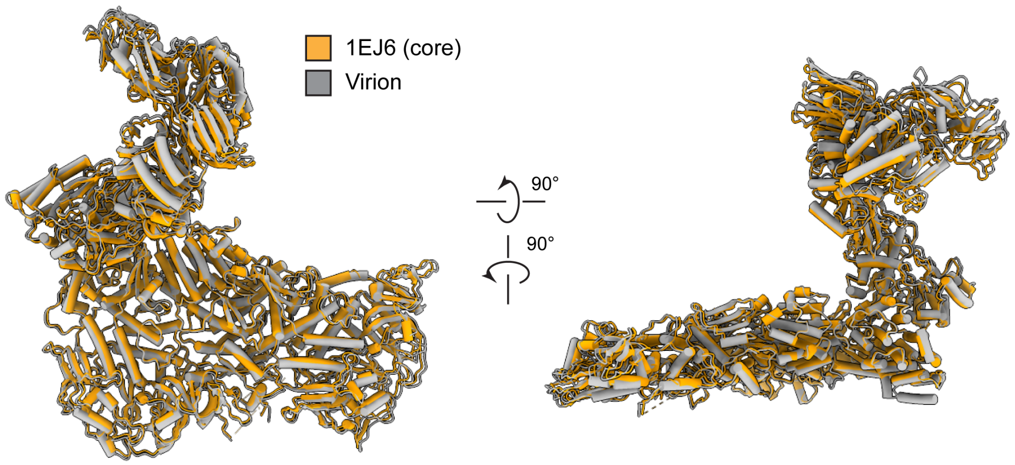


**Fig. S6: Structure superposition of λ2 from core (published crystal structure) and virion (reported in this study).** The two structures are almost identical but different from the cryoEM structure of MRV core isolated from cell cytoplasm reported in this study.

## Supplementary Table

### Table S1. Summary for CryoET data collection and subtomogram averaging of cells at 6 h post-infection

| | Star core | Core<br>EMD-47313 | Empty core<br>EMD-47314 | Virion | Empty virion | Outer shell |
|---|---|---|---|---|---|---|
| **Magnification** | | | 42,000 | | | |
| **Voltage (kV)** | | | 300 | | | |
| **Pixel size (Å)** | | | 1.965 | | | |
| **Total dose ($e^-$/Å$^2$)** | | | 100 | | | |
| **Defocus range (μm)** | | | -3.5 to -5 | | | |
| **Acquisition scheme** | | | Dose symmetric scheme<br>-60° - 40° or -40° - 60° | | | |
| **Tilt series number** | | | 20 | | | |
| **Symmetry imposed** | I3 | I3 | I3 | I3 | I3 | I3 |
| **Particle number** | 23 | 13 | 22 | 125 | 13 | 23 |
| **Map resolution (Å)** | 20 | 16 | 20 | 16 | 34 | 38 |
| **FSC threshold** | 0.143 | 0.143 | 0.143 | 0.143 | 0.143 | 0.143 |

**Table S2. Summary for CryoET data collection and subtomogram averaging of cells at 48 h post-infection**

| | Star core | Core | Empty core | Virion | Empty virion | Outer shell |
|---|---|---|---|---|---|---|
| | EMD-47315 | | | EMD-47316 (EMD-47317) | EMD-47318 | EMD-47319 |
| **Magnification** | | | 33,000 | | | |
| **Voltage (kV)** | | | 300 | | | |
| **Pixel size (Å)** | | | 2.6 | | | |
| **Total dose ($e^-$/Å$^2$)** | | | 100 | | | |
| **Defocus range (µm)** | | | -3.5 to -5 | | | |
| **Acquisition scheme** | | | Dose symmetric scheme<br>-60° - 40° or -40° - 60° | | | |
| **Tilt series number** | | | 25 | | | |
| **Symmetry imposed** | I3 | I3 | I3 | I3 (C5)* | I3 (C5)* | I3 |
| **Particle number** | 67 | 29 | 32 | 1292 (11,968) | 432 (2,470) | 43 |
| **Map resolution (Å)** | 14 | 27 | 19 | 13 (6.9) | 17 (10.9) | 40 |
| **FSC threshold** | 0.143 | 0.143 | 0.143 | 0.143 | 0.143 | 0.143 |

*Numbers in the parentheses are results from sub-particle reconstructions.

**Table S3. CryoEM data collection, refinement and validation statistics of MRV isolate from cells at 6 h post-infection**

| | Core | Unturreted empty core | Turreted empty core | Virion | Empty virion |
|---|---|---|---|---|---|
| **EMDB** | **EMD-46053** | **EMD-47321** | **EMD-47322** | **EMD-46054** | |
| **PDB** | **9CYX** | | | **9CYY** | |
| **Data collection and processing** | | | | | |
| Magnification | | | 81,000 | | |
| Voltage (kV) | | | 300 | | |
| Electron exposure ($e^-$/Å$^2$) | | | 50 | | |
| Defocus range (μm) | | | -1.8 to -2.6 | | |
| Pixel size (Å) | | | 1.1 | | |
| Symmetry imposed | C5 | C5 | C5 | C1 | C5 |
| Particle number | 10,857 | 26,781 | 8,093 | 97,187 | 60,524 |
| Map resolution (Å) | 3.3 | 3.6 | 3.8 | 3.0 | 3.9 |
| FSC threshold | 0.143 | 0.143 | 0.143 | 0.143 | 0.143 |
| **Refinement** | | | | | |
| Model resolution (Å) | 3.5 | | | 3.0 | |
| FSC threshold | 0.5 | | | 0.5 | |
| Model composition | | | | | |
| Non-hydrogen atoms | 34529 | | | 145753 | |
| Protein residues | 4377 | | | 18565 | |
| Nucleotide | 0 | | | 0 | |
| *B* factors (Å$^2$) | | | | | |
| Protein | 32 | | | 110 | |
| Ligand | | | | | |
| R.m.s. deviations | | | | | |
| Bond lengths (Å) | 0.006 | | | 0.003 | |
| Bond angles (°) | 0.746 | | | 0.587 | |
| Validation | | | | | |
| MolProbity score | 1.63 | | | 1.41 | |
| Clashscore | 6.01 | | | 5.33 | |
| Poor rotamers (%) | 0.34 | | | 0.14 | |
| Ramachandran plot | | | | | |
| Favored (%) | 95.66 | | | 97.39 | |
| Allowed (%) | 4.34 | | | 2.61 | |
| Disallowed (%) | 0.00 | | | 0 | |

**Table S4. CryoEM data collection, refinement and validation statistics of MRV isolate from cells at 48 h post-infection**

| | Virion | Empty virion | Outer shell |
|---|---|---|---|
| **EMDB** | | **EMD-47320** | **EMD-46049** |
| **PDB** | | | **9CYT** |
| **Data collection and processing** | | | |
| Magnification | | 81,000 | |
| Voltage (kV) | | 300 | |
| Electron exposure ($e^-/Å^2$) | | 50 | |
| Defocus range (μm) | | -1.8 to -2.6 | |
| Pixel size (Å) | | 1.1 | |
| Symmetry imposed | C5 | C5 | C5 |
| Particle number | 67,928 | 22,373 | 8,911 |
| Map resolution (Å) | 3.0 | 3.2 | 3.7 |
| FSC threshold | 0.143 | 0.143 | 0.143 |
| **Refinement** | | | |
| Model resolution (Å) | | | 3.6 |
| FSC threshold | | | 0.5 |
| Model composition | | | |
| Non-hydrogen atoms | | | 33997 |
| Protein residues | | | 4390 |
| Nucleotide | | | 0 |
| *B* factors ($Å^2$) | | | |
| Protein | | | 14.7 |
| Ligand | | | |
| R.m.s. deviations | | | |
| Bond lengths (Å) | | | 0.004 |
| Bond angles (°) | | | 0.621 |
| Validation | | | |
| MolProbity score | | | 1.62 |
| Clashscore | | | 6.22 |
| Poor rotamers (%) | | | 0.16 |
| Ramachandran plot | | | |
| Favored (%) | | | 96.02 |
| Allowed (%) | | | 3.98 |
| Disallowed (%) | | | 0 |
