# Supplementary material for: Molecular sociology of virus-induced cellular condensates supporting reovirus assembly and replication
Source: Nat Commun. 2024 Dec 6;15:10638. doi: 10.1038/s41467-024-54968-7 (PMC11621325; doi:10.1038/s41467-024-54968-7)
Supplement: Supplementary file 3 — Description Of Additional Supplementary File [file 41467_2024_54968_MOESM3_ESM.pdf]

Description of Additional supplementary files

Video S1.

Description: Slices through cryoET reconstruction and the 3D segmentation of tomogram shown in Fig. 1c-d (scale bar: 100 nm).

Video S2.

Description: Slices through cryoET reconstruction and the 3D segmentation of tomogram shown in Fig. 1e-f (scale bar: 100 nm).

Video S3.

Description: Slices through cryoET reconstruction and the 3D segmentation of tomogram shown in Fig. 2a-b (scale bar: 100 nm).

Video S4.

Description: Propagation of inner capsid at 5-fold axis from star core to core during virus assembly.

Video S5.

Description: Conformational changes of λ2 from core to virion during virus assembly
